# An audit assessing the monitoring of SSRIS after initiation in children and adolescents

**DOI:** 10.1192/bjo.2021.159

**Published:** 2021-06-18

**Authors:** Ella McGowan

**Affiliations:** Black Country Healthcare

## Abstract

**Aims:**

To identify children and adolescents started on SSRIs to see if they are being followed up in accordance to NICE and Maudsley guidelines

Objectives

Has the patient been followed up after a week to check for adverse effects or improvement in their mental state?

Has the patient been re-evaluated every 4-6 weeks, if not is there an alternative plan?

If there is no improvement has the dose been increased?

If there is an adverse effect has the dose been lowered or the medication stopped?

**Method:**

Paper case notes including clinic letters and handwritten notes were reviewed on the 19/10/2020. The following data were collected anonymously.

Age

Gender

Date seen / Date medication started

Name of medication

Date medication started

Date of Follow-up

Monitoring of improvement

Monitoring of adverse effects

Outcome of monitoring

**Result:**

A total of 18 sets of cases were identified.

Follow-up occurred in 17 of the 18 cases.

The one case that had not been followed up had started the medication 8 weeks before the audit. The median follow-up time was 42 days (6 weeks). No cases were followed up within a week.

Monitoring of improvement was recorded in 88% of case notes reviewed.

Monitoring for adverse effects occurred in 36% of case notes and none of these patients had reported any side effects. 53% of cases did not have monitoring of adverse effects documented. There were two patients (11%) who did not take the medication as prescribed. One out of choice and one their parent had not collected it.

The medication dose was increased in 22% of patients without clear documentation of monitoring for adverse effects.

**Conclusion:**

After discussion with the clinical lead it was decided it is impractical to follow up patients a week after starting medication. However, patients and their carers should be informed of the side effects and advised to contact CAMHS if adverse effects occur.

The area of practice that can be improved is the documentation of adverse effects at follow-up.

Recommendations:

All patients to be informed of the common side effects of the medication before it is initiated and advised to contact the CAMHS team if they have concerns

All CAMHS patients started on SSRIs should be followed up within 4-6 weeks

At follow-up any adverse events and clinical response should be discussed

An accurate record of the exchanges of the above information should be documented in the notes

Re-audit

